# Genotoxicity and cytotoxicity of cone beam computed tomography in children

**DOI:** 10.1186/s12903-021-01792-w

**Published:** 2021-09-04

**Authors:** Doua H. Altoukhi, Sumer Alaki, Eman El Ashiry, Osama Nassif, Dania Sabbahi

**Affiliations:** 1 Department of Pediatric Dentistry, Faculty of Dentistry, King Abdulaziz University, Jeddah, Saudi Arabia; 2 Department of Pathology, Faculty of Medicine and Hospitals, King Abdulaziz University, Jeddah, Saudi Arabia; 3 Department of Dental Public Health, Faculty of Dentistry, King Abdulaziz University, Jeddah, Saudi Arabia

**Keywords:** Cone beam computed tomography, Micronucleus, Children, Buccal mucosa

## Abstract

**Background:**

Dental radiographs are essential tools for diagnosis. However, there are significant concerns about the dangerous effect of radiation especially on children. The aim of this study was to evaluate genotoxicity and cytotoxicity in the exfoliated cells of buccal mucosa of children subjected to Cone Beam Computed Tomography (CBCT).

**Methods:**

The study included 18 healthy children aged (9–12 years) who were exposed to CBCT. All CBCT scans were performed with the i-CAT CBCT. Exfoliated buccal cells were scraped from the left and right cheek immediately before the exposure, after 10 ± 2 days, and after 1 month. Cells were stained using Feulgen/fast green stain and examined under light microscopy. Genotoxicity (Micronuclei) and cytotoxicity (condensed chromatin, karyorrhexis, pyknosis, and karyolysis) were scored. Statistical analysis was performed using the McNemar test, Wilcoxon Signed-Rank test, and Mann-Whitney U test at a significance level of *p* < 0.05.

**Results:**

There were statistically significant differences in the mean percentages of micronuclei, condensed chromatin, karyorrhexis, pyknosis, and karyolysis before and 10 ± 2 days after the CBCT scan (*p* < 0.05). There were no statistically significant differences in the frequency of micronuclei, condensed chromatin, karyorrhexis, or pyknosis before and 1 month after the exposure (*p* > 0.05) except for karyolysis (*p* < 0.05).

**Conclusions:**

CBCT may induce genotoxicity and cytotoxicity in buccal mucosa cells of children. Therefore, CBCT should not be prescribed unless necessary as it cannot be considered a risk-free procedure.

## Background

Radiographs help dental practitioners definitively diagnose oral conditions that cannot be diagnosed by clinical examination alone [1, 2]. Recently, Cone Beam Computed Tomography (CBCT) is recognized as an encouraging radiographic technique that has been utilized in different dental specialties such as dentomaxillofacial radiologists, orthodontics, periodontics, and endodontics [3–7]. CBCT can provide a three-dimensional (3D) image of oral structures and a sharp image of high contrast structures, such as bones [8].

Although dental radiographs are essential tools for diagnosis [1], there are significant concerns about the dangerous effect of radiation. Children are at higher risk from radiation than adolescents and adults because (1) the fast-growing tissues in children are considered more radiosensitive than mature tissues in adults; (2) a child has a longer life expectancy compared to an adult; thus, the cumulative radiation effect has more extended periods to cause cancers; (3) effective dose of CBCT radiation, which is the measurement of the harmful effect of radiation to the human body, is approximately 30% greater in children than in adolescents; (4) the organ dose for children, which is the absorbed dose to a particular organ, is greater than the adolescents with the salivary glands getting a more significant dose compared to other head and neck organs. The thyroid also is more affected in children due to its smaller size [9] and (5) radiation dose for a child may exceed an adult radiation dose unless specific exposure-reduction protocols for children are incorporated. Appropriate field of view (FOV) selection that matches the interest area provides a considerable dose saving [10]. In conclusion, children could have up to ten times higher liability to radiation-induced carcinogenesis than adults [11–14].

Radiation can produce damage in different pathways: chromosomal damage (genotoxicity) leading to micronucleus (MN) formation [15] or (cytotoxicity) represented as nuclear changes other than micronucleus leading to cell death [16]. Micronucleus (MN) is characteristically seen in the exfoliated epithelial cells such as buccal mucosa during cancerous and precancerous conditions [17]. Micronuclei are cytoplasmic chromatin masses that appear as small nuclei arise from acentric chromosome fragments or lagging chromosomes during the transition from metaphase to anaphase of mitosis [15, 18]. The turnover of oral epithelium is fast (7–16 days), and therefore micronuclei reflect genotoxic insults that occurred 1–3 weeks earlier in the basal dividing layer [19–22]. Some of the basal cells might degenerate into cells with condensed chromatin, shrunken high-density nuclei (pyknosis), fragmented nuclei (karyorrhexis), or completely lose their nuclei (karyolysis) as explained by Tolbert et al. in 1992 [23].

Exfoliated buccal cells, which are considered the area target for most dental radiographs, have been noninvasively and successfully used to show the cytotoxic and genotoxic effects of radiation [24, 25]. Only a few studies investigate the genotoxic and cytotoxic outcomes of CBCT on children’s buccal mucosa. In 2010, Carlin et al. found no significant differences in the micronucleus rate (genotoxicity) preceding and 10 days following CBCT. However, the study found that CBCT led to a significant increase in the other nuclear alterations (cytotoxicity) such as karyolysis, karyorrhexis, and pyknosis [26]. Another study by Lorenzoni et al. in 2013 examined the genotoxicity and cytotoxicity of CBCT compared to orthodontic Radiographic Set. The results showed that CBCT leads to more increase in cytotoxicity [27]. Li et al. in 2018 revealed a significant difference in the micronucleus rate before and after exposure to one or more of the following X-rays: panoramic radiograph, cephalometric radiograph, and CBCT [28].

As seen from the previous studies, growing children have an increased susceptibility to the harmful effect of radiation. However, none of the studies did further follow-up after approximately 10 days following CBCT exposure. This fact indicates the paramount need to investigate the residual effect from radiation and the extent of damage on the buccal mucosa over a more extended period.

Therefore, the current study aims to evaluate genotoxicity in terms of micronuclei and cytotoxicity in terms of condensed chromatin, karyorrhexis, pyknosis, and karyolysis among children’s exfoliated buccal cells who were subjected to CBCT over the following periods: 10 ± 2 days and 1 month after exposure; and compare them to the baseline.

## Methods

### Study design

This study is a prospective cohort study that evaluates participants over different periods: baseline, 10 ± 2 days, and 1 month after CBCT exposure. Research approval was obtained from the Research Ethics Committee of KAUFD under ethical approval number 145-11-18.

### Sample

Twenty healthy (ASA I) children aged between 9 and 12 years, with fair oral hygiene according to the Greene and Vermillion Simplified Oral Hygiene Index, were not exposed to any head and neck radiation within the last six months, were included in our study.

Participants who had justified clinical indication of mesioangular maxillary permanent canines which had a tendency for impaction, as seen from their panoramic radiographs taken in the last 6–12 months, were enrolled from the Pediatric Dentistry Clinic at University Dental Hospital at King Abdulaziz University, Jeddah, Saudi Arabia. The sample size was calculated for the medium effect to be 10 participants using G*Power software (Version 3.1.9.3) (HHU, Germany) at 80% power and 0.05 significance level. The sample size was increased to 20 participants in order to compensate for any dropout. Twenty children with signed informed consent from their legal guardians were included in the study.

#### CBCT scanning protocol

The CBCT requests were made by a pediatric dentst. The images were taken with an i-CAT CBCT scanner (Kavo Kerr, United States) at the Oral Radiology Department to have a 3D image of the exact position of the unerupted maxillary permanent canines. This scanner was used only on the maxillary region. The following parameters were used for the CBCT: FOV 16 × 6 cm, 120 kV, 10 mA, 4.8 s and 0.4 voxels. The total effective dose was around 22 µSv.

### Buccal mucosal cell collection

Before buccal cells collection, children were instructed to wash their mouths with water thoroughly to eliminate debris. Exfoliated buccal cells were taken from every child by scraping the buccal mucosa on both right and left sides with a Rovers ® special brush (BD, Netherlands) immediately before radiographic exposures (baseline), after 10 ± 2 days [27–31] and after 1 month. Then, the scraped cells were collected in sample bottles that contain BD SurePath™ Preservative Fluid: ethanol, methanol, and isopropanol (BD, Ireland).

### Cytological preparations and scoring

Cytological preparations were performed in the Cytology lab, King Abdulaziz University Hospital (KAUH). The collected samples were placed in a centrifuge (3400 rpm) for 3 min (Hettich, Germany). Then, the supernatant layer was removed. One thousand cells were taken from each sample using a manual cell counting chamber (Lafontaine, Belgium) to be stained. The cells were placed on a charge slide (Thermo Scientific, United States). The slides were then fixed for 20 min in 95% Ethanol (Honeywell, United States). Cytological preparations were then stained with a DNA-specific stain named Feulgen/fast green (Bio-Optica, Italy) and examined under light microscopy (Olympus, Japan) at x400 magnification [27–29]. Genotoxicity (micronuclei) and cytotoxicity (karyolysis, pyknosis, condensed chromatin, and karyorrhexis) were scored following the criteria explained by Tolbert et al. in 1992 [23].

Each slide was evaluated for the presence of:

1. Micronucleus.

Cells with micronuclei can be described by the existence of the main nucleus with another smaller nucleus or nuclei. A MN must (i) have similar refraction, texture, and color to the main nucleus; (ii) be an oval or a round in shape (iii) be smaller than one-third of the main nucleus; (iv) be located in the cell cytoplasm; (v) be located on the main nucleus plane of focus; (vi) be visibly separated from the main nucleus [23].

2. Condensed chromatin.

Condensed chromatin cells show nuclei with aggregated chromatin regions revealing a speckled nuclear pattern. The chromatin is accumulating in some areas of the nucleus, whereas it is disappeared in other areas.

3. Karyorrhexis.

More extensive chromatin aggregation is seen in the nucleus of karyorrhectic cells, which causes degeneration and fragmentation of the nucleus.

4. Pyknosis.

The pyknotic cells are presented with shrunken nuclei that have high-density and uniformly stained nuclear material.

5. Karyolysis.

Karyolytic cells are completely diminished of DNA in the nucleus with no Feulgen staining leading to the ghost-like appearance of the cell [23].

### Cytological analysis

Buccal cells from each patient were investigated preceding and following X-ray exposure. Cytological observations were accomplished using a light microscope (Olympus, Japan) at x400 magnification attached to a digital camera (SC 180) (Olympus, Japan) at the Cytology lab, KAUH. The frequencies of micronuclei and the other nuclear changes were counted in 1000 cells for each individual in each follow-up period [27–29]. The microscope was attached to a computer during this period. Thus, the cells can be scored easily on the large screen using cellSens imaging software (Olympus, Japan).

All slides were examined by a well-trained and calibrated pediatric dentist and frequently rechecked by a second examiner, a blinded pathologist. The second examiner did not know who the patients are and when the cells were obtained. Before starting, a pilot study was done to measure the intra-examiner and inter-examiner reliability after training using the Kappa test. Sixteen slides from four patients (four slides for each patient) were scored by the pediatric dentist. The same slides were examined two weeks later by the same pediatric dentist for intra-examiner variances and the pathologist for inter-examiner variances.

### Statistical analysis

McNemar test was used to compare the percentages of subjects with genotoxic changes and cytotoxic changes at the three different periods. Wilcoxon Signed-Rank test was applied to compare the mean percentages of genotoxicity (micronuclei) and cytotoxicity (condensed chromatin, karyorrhexis, pyknosis, and karyolysis) at the three different periods. Moreover, Mann-Whitney U test was used to compare the mean percentages of genotoxicity changes and cytotoxicity changes between males and females at each of the three periods. The level of significance was fixed at *p* < 0.05. SPSS version 22 (IBMCorp., 2017) was used to accomplish the statistical analysis.

## Results

A total of 20 individuals were enrolled in this study. Two of the participants were lost to follow-up after the first swap, so they were excluded. Thus, the remaining sample was 18 (8 males and 10 females). Out of the 18 participants, two missed their 1-month follow-up, and one lost his 10 ± 2 days follow-up. The mean age was 11 ± 1.0 (10.7 ± 0.7 years for the male subjects and 11.3 ± 1.1 years for the female subjects) (Table 1).

**Table. 1:** Demographic data of the study participants

Gender	Female	Male
Number of participants	10	8
Mean age (years) ± SD	11.3 ± 1.1	10.7 ± 0.7

According to the Kappa test, the inter-examiner reliability was 0.88, and the intra-examiner reliability was 0.87, which indicates excellent agreement.

The percentages of subjects presented with micronuclei were 80% after 10 ± 2 days and 56% after 1 month following the exposure, which was higher than the percentage before the exposure 50%. Moreover, the rates of subjects presented with other nuclear changes (condensed chromatin, karyorrhexis, and karyolysis) at 10 ± 2 days and 1 month following CBCT exposure were higher than the baseline percentages. However, the differences were not statistically significant (*p* > 0.05) (Table 2).

The mean percentage of micronuclei before the exposure was 0.3 ± 0.3, which was significantly increased to 2.2 ± 2.3 after 10 ± 2 days since CBCT exposure (*p =* 0.00). The mean percentage of micronuclei continued to decrease until it reached 0.1 ± 0.1 after 1 month from the exposure, which showed no statistically significant difference than the baseline (*p* = 0.25). Besides, a statistically significant increase was observed in the mean percentages of all other nuclear changes 10 ± 2 days following the exposure: condensed chromatin (*p =* 0.04), karyorrhexis (*p =* 0.01), pyknosis (*p =* 0.01), and karyolysis (*p =* 0.00). The mean percentages of other nuclear changes decreased 1 month after the exposure. Compared to the baseline, the mean rates of condensed chromatin (*p =* 0.18), pyknosis (*p =* 0.61), and karyorrhexis (*p =* 0.17) were not significantly different. However, karyolysis showed a statistically significant increase compared to the baseline (*p* = 0.01) (Table 3).

**Fig. 1:**
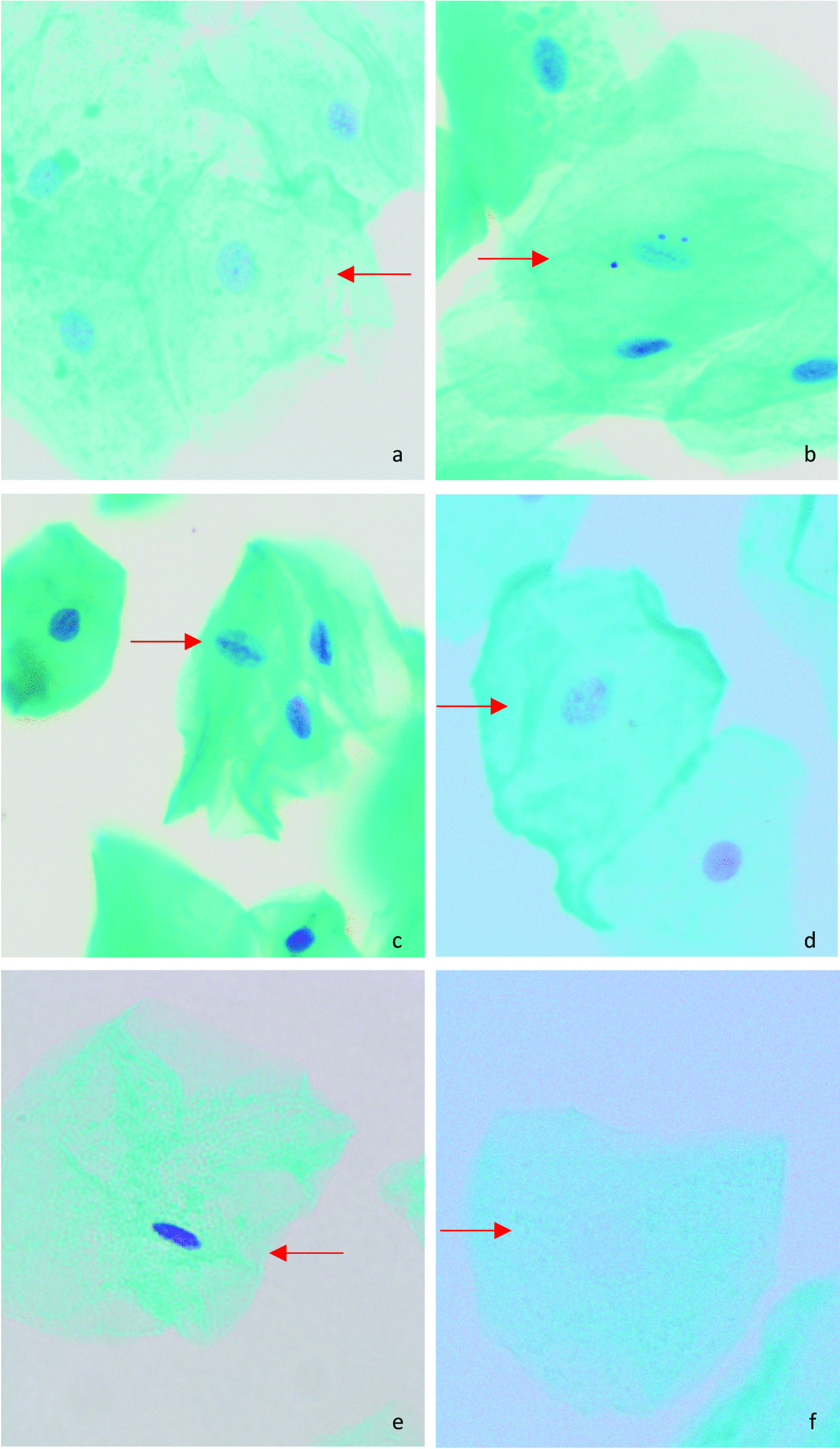
Microscopic views of buccal cells (Feulgen/fast green stain, x400 magnification): **a** normal cell, **b** micronucleated cell, **c** condensed chromatin cell, **d** Karyorrhectic cell **e** pyknotic cell, **f** Karyolytic cell (arrow)

There were no statistily significant differences between males and females regarding the mean percentages of micronuclei, condensed chromatin, karyorrhexis, pyknosis, or karyolysis in the three different periods: baseline, 10 ± 2 days, and 1 month after CBCT exposure (*p* > 0.05) (Table 4). Figure 1 shows an example of a micronucleated cell and other nuclear changes.

**Table 2:** Percentage of subjects with genotoxic changes (micronucleus) and cytotoxic changes (condensed chromatin, karyorrhexis, pyknosis, and karyolysis)

Cellular Alterations	Baseline	10 days		1 month	
	Percentage	Percentage	***p***-value^a^	Percentage	***p***-value^a^
Micronucleus	50%	82%	0.13	56%	0.69
Condensed chromatin	83%	88%	1.00	93%	0.63
Karyorrhexis	33%	59%	0.13	50%	0.51
Pyknosis	100%	100%	NA**	100%	NA**
Karyolysis	77%	94%	0.25	88%	0.50

a. McNemar test

**McNemar test is not applicable

**Table 3:** Mean percentage of cells with genotoxic changes (micronucleus) and cytotoxic changes (condensed chromatin, karyorrhexis, pyknosis, and karyolysis)

Cellular alterations	Baseline		10 days			1 month		
	Mean ± SD	Median	Mean ± SD	Median	*p-*value^a^	Mean ± SD	Median	*p*-value^a^
Micronucleus	0.3 ± 0.3	0.1	2.2 ± 2.3	1.6	0.00*	0.1 ± 0.1	0.1	0.25
Condensed chromatin	0.9 ± 0.7	0.9	1.4 ± 0.9	1.6	0.04*	1 ± 0.7	1.1	0.18
Karyorrhexis	0.1 ± 0.1	0.0	0.2 ± 0.2	0.2	0.01*	0.1 ± 0.2	0.1	0.17
Pyknosis	9.9 ± 7.7	9.2	18.3 ± 10.7	15.7	0.01*	9.8 ± 8.1	9.2	0.61
Karyolysis	0.4 ± 0.4	0.4	1.7 ± 1.6	1.2	0.00*	1.2 ± 0.9	1.0	0.01*

a. Wilcoxon Signed-Rank test

*p is statistically significant at > 0.05

**Table 4:** Mean percentage of cells with genotoxic changes (micronucleus) and cytotoxic changes (condensed chromatin, karyorrhexis, pyknosis, and karyolysis) among males and females

Cellular alterations	Baseline				10 days			1 month		
	Mean ± SD		Median	*p*-value^a^	Mean ± SD	Median	*p*-value^a^	Mean ± SD	Median	*p*-value^a^
Micronucleus	F	0.2 ± 0.3	0.1	0.74	2.8 ± 2.3	1.8	0.18	0.1 ± 0.2	0.1	0.47
	M	0.3 ± 0.3	0.2		1.5 ± 2.2	0.5		0.1 ± 0.1	0.1	
Condensed chromatin	F	1.00 ± 0.8	0.9	0.42	1.5 ± 0.8	1.6	0.53	1 ± 0.7	0.8	0.56
	M	0.7 ± 0.6	0.8		1.3 ± 1.0	1.2		1.1 ± 0.6	1.2	
Karyorrhexis	F	0.1 ± 0.1	0.0	0.87	0.1 ± 0.2	0.0	0.42	0.1 ± 0.1	0.0	0.31
	M	0.1 ± 0.1	0.0		0.2 ± 0.2	0.2		0.2 ± 0.3	0.1	
Pyknosis	F	8.3 ± 3.9	9.2	0.59	13.6 ± 7.1	12.9	0.10	9.2 ± 5.1	10.2	0.53
	M	11.9 ± 10.8	9.9		23.6 ± 11.9	26.6		10.4 ± 10.7	7.7	
Karyolysis	F	0.3 ± 0.4	0.3	0.10	1.2 ± 1.5	0.6	0.16	1.1 ± 0.9	0.9	0.60
	M	0.6 ± 0.4	0.5		2.3 ± 1.6	2.5		1.4 ± 0.9	1.3	

a. Mann-Whitney U test

F. Females.

M. Males.

## Discussion

Oral cancer is considered the sixth most common world cancer [32], and about 90% of human oral cancers originate from epithelium [33]. Those crucial facts emphasize the benefit of the micronucleus test, which is an in vivo test revealing the direct effect of toxic agents on the target tissues such as buccal mucosa. The relative simplicity of scoring, little cost, and accuracy attained by scoring a significant number of cells increase the acceptance of this non-invasive technique [34].

Genotoxic damages, which can cause micronuclei formation, occur in the basal cell layer of epithelium, where cells experience mitosis. These basal cells move to the surface layer and undergo exfoliation due to the fast turnover of epithelium [35]. Epithelial cells need approimately 7–16 days to reach the surface layer and then exfoliate [36]. Therefore, exfoliated buccal mucosa cells in the current study were scraped immediately before the exposure to ionizing radiation and 10 ± 2 days after. The 1-month follow-up period was chosen to examine the residual genotoxic and cytotoxic effects of the radiation on epithelial cells. The oral epithelium depends on stem cells for regeneration [37]. It is well-known that the stem cells of normal tissues establish a life-long reservoir of cells’ ability for self-regeneration. The division of epithelial cells occurs mainly in the basal layer that encloses stem cells. Nevertheless, tissue regeneration and maintenance mechanisms of these cells are still unknown [38], and they could be affected by the radiation.

Human biomonitoring studies in the buccal mucosa, in which the human tissues are examined for contamination with contaminants away from the test person, involve many confounding factors such as age, oral hygiene, dental health, smoking, viruses, and alterations in the immune system [39]. In the current study, these confounding factors were controlled. The sample included only healthy subjects between 9 and 12 years of age with fair oral hygiene. Young individuals are usually less vulnerable to confounding factors such as occupational exposure and cigarette smoking than adults [40]. Besides, every child was served as his control. Hence, the effect of any other genotoxic agent should have been existing in the first sample. Consequently, the variation between the three samples can be attributed to the radiation [41].

The percentages of children presented with genotoxicity (micronuclei) and cytotoxicity (condensed chromatin, karyorrhexis, and karyolysis) 10 ± 2 days and 1 month after radiation exposure are always higher than the baseline. This increase confirms the genotoxic and cytotoxic effects of CBCT among children.

After the CBCT scan by 10 ± 2 days, the mean percentages of micronuclei were significantly increased, indicating that CBCT can induce a genotoxic effect on the buccal mucosa of children. This finding is in line with the results from the study of Fonte et al., which was performed on adults to investigate the genotoxicity and cytotoxicity of CBCT in oral exfoliated cells and confirmed that CBCT offers the risk of inducing genetic damage [42]. These results contrast with the findings of another study performed on children to compare mutagenicity and cytotoxicity in exfoliated buccal cells after CBCT exposure and reported that micronuclei were not significantly increased 10 ± 2 days after the exposure [43]. Findings of the current study also oppose the results of Carlin et al. [44] and Yang et al. [29] which were performed on adults. Biomonitoring studies of individuals subjected to radiation are relatively complex and non-specific as there are variations in the radiation dose in each study population. These challenges can justify why several studies found a high level of genetic damage in their study individuals after radiographic exposure and some did not. Moreover, the characteristics of the population and the methodological aspects like differences in swaps sites, collection of cells, the fixing techniques, staining procedures, number of counted cells, and micronucleus scoring criteria might affect the results [39].

Researchers have raised attention to the nuclear alterations other than micronucleus, which illustrate cellular death and might improve the tests’ sensitivity to detect genotoxicity [23, 45]. Therefore, the cytotoxic effect was examined through the incidences of condensed chromatin, karyorrhexis, pyknosis, and karyolysis. CBCT stimulated cellular death, as shown by the significant changes between values before and 10 ± 2 days after the exposure (*p* < 0.05), in agreement with the results of other studies [29, 42–44]. These findings reinforce the idea that CBCT might promote cytotoxicity in the buccal mucosa.

Statistics of this study showed no statistically significant differences between the values before and 1 month following CBCT exposure, neither for the micronucleus nor condensed chromatin, karyorrhexis, and pyknosis (*p* > 0.05). However, karyolysis showed a statistically significant increase after one month compared to the baseline (*p* < 0.05). These findings indicate that CBCT still has a cytotoxic effect on the buccal mucosa of children after 1 month. Karyolysis may show a very late stage in cell death (necrosis), suggesting that CBCT does produce a cytotoxic effect that could lead to necrosis [46]. Researches revealed that pyknosis and condensed chromatin are considered common associates of epithelial cell maturation and differentiation. Though, they indicate cellular injury if they occur at elevated levels. Karyorrhexis, pyknosis, and condensed chromatin show early stages of apoptosis, which is considered the primary type of cell death in living tissues. It is known as a programmed cell death that occurs through normal cell turnover and under physiological control [47, 48]. Apoptosis may have a surveillance role, eradicating cells with genetic damage stimulated by chemicals that bond Deoxyribonucleic acid (DNA) or ionizing radiation. Therefore, apoptosis over normal levels is indicative of genotoxic insult [23].

The results of the present study show an extended genotoxic and cytotoxic effects from CBCT radiation on the buccal mucosa of children 1 month following the exposure, although it is not significant except for karyolysis. These results are of great value as none of the previous studies did further follow-up after 10 ± 2 days following CBCT exposure. Thus, these facts suggest further research with a more extended follow-up period and a larger sample to inspect the residual effect of radiation.

## Conclusions

The present study indicates that CBCT could induce cytotoxic and genotoxic effects on buccal mucosa cells of children. Therefore, CBCT should only be prescribed when necessary as it cannot be considered a risk-free procedure. Further studies on children with a larger sample size and a longer follow-up period are recommended.

## Data Availability

The dataset analyzed in the current study is available upon reasonable request from the corresponding author.

## Abbreviations

CBCT: Cone beam computed tomography
3D: Three-dimensional
FOV: Field of view
MN: Micronucleus
KAUH: King Abdulaziz University Hospital
DNA: Deoxyribonucleic acid
F: Female
M: Male
